# Comparative Analyses of Chloroplast Genomes From 14 *Zanthoxylum* Species: Identification of Variable DNA Markers and Phylogenetic Relationships Within the Genus

**DOI:** 10.3389/fpls.2020.605793

**Published:** 2021-01-13

**Authors:** Kaihui Zhao, Lianqiang Li, Hong Quan, Junbo Yang, Zhirong Zhang, Zhihua Liao, Xiaozhong Lan

**Affiliations:** ^1^TAAHC-SWU Medicinal Plant Joint R&D Center, Tibetan Collaborative Innovation Center of Agricultural and Animal Husbandry Resources, Food Science College, Tibet Agriculture and Animal Husbandry University, Nyingchi, China; ^2^Key Laboratory of Forest Ecology in Tibet Plateau, Tibet Agricultural and Animal Husbandry University, Ministry of Education, Nyingchi, China; ^3^Germplasm Bank of Wild Species, Kunming Institute of Botany, Chinese Academy of Sciences, Kunming, China; ^4^Key Laboratory of Eco-Environments in the Three Gorges Reservoir Region, Ministry of Education, Chongqing Engineering and Technology Research Center for Sweetpotato, School of Life Sciences, Southwest University, Chongqing, China

**Keywords:** *Zanthoxylum*, chloroplast genome, divergent hotspots, DNA barcode sequence, phylogeny

## Abstract

*Zanthoxylum* L. is an economic crop with a long history of cultivation and domestication and has important economic, ecological, and medicinal value. To solve the classification problems caused by the similar morphological characteristics of *Zanthoxylum* and establish a credible phylogenetic relationship, we sequenced and annotated six *Zanthoxylum* chloroplast (cp) genomes (*Z. piasezkii, Z. armatum, Z. motuoense, Z. oxyphyllum, Z. multijugum*, and *Z. calcicola*) and combined them with previously published genomes for the *Zanthoxylum* species. We used bioinformatics methods to analyze the genomic characteristics, contraction, and expansion of inverted repeat (IR) regions; differences in simple sequence repeats (SSRs) and long repeat sequences; species pairwise Ka/Ks ratios; divergence hotspots; and phylogenetic relationships of the 14 *Zanthoxylum* species. The results revealed that cp genomes of *Zanthoxylum* range in size from 158,071 to 158,963 bp and contain 87 protein-coding, 37 tRNA, and 8 rRNA genes. Seven mutational hotspots were identified as candidate DNA barcode sequences to distinguish *Zanthoxylum* species. The phylogenetic analysis strongly supported the genus *Fagara* as a subgenus of *Zanthoxylum* and proposed the possibility of a new subgenus in *Zanthoxylum.* The availability of these genomes will provide valuable information for identifying species, molecular breeding, and evolutionary analysis of *Zanthoxylum.*

## Introduction

*Zanthoxylum* L. belongs to the *Rutoideae* subfamily of the *Rutaceae* family. *Zanthoxylum* is widely distributed in tropical and subtropical regions and consists of approximately 250 species (Huang, 1997). Owing to its important economic and medicinal value, *Zanthoxylum* has a long history of cultivation and domestication in Asia. The Chinese pharmacopeia (2015 Edition) records that the dried and mature peel of *Zanthoxylum schinifolium* Sieb. et Zucc. and *Zanthoxylum bungeanum* Maxim. have the effects of warming the middle-jiao to alleviate pain, destroying parasites, and relieving itching, and can be used to treat cold pain of the gastric cavity and abdomen, vomiting and diarrhea, and abdominal pain due to parasitic infestation. The pericarp of *Z. armatum, Z. bungeanum*, and other *Zanthoxylum* species has a strong fragrance and is one of the traditional Chinese “eight major condiments.” In addition, owing to its economic potential, *Zanthoxylum* has been planted to restore farmland to forests and has high ecological value for soil and fertilizer preservation (Hu et al., 2012).

Due to the high medicinal and culinary value of the *Zanthoxylum* genus plants, there are some phenomena of homonym, heteronym, or defective products that are substituted for the qualified ones in the market (Shen et al., 2004). The traditional classification and identification of *Zanthoxylum* relied on its morphological characteristics, such as leaf morphology, fruit color, fruit maturity, leaf gland points, and leaf thorns (Tu et al., 2001), however, the morphological characteristics are easily affected by the growth environment. Also, many *Zanthoxylum* plants are similar in morphology and difficult to identify, which restricts the progress and development of the *Zanthoxylum* industry. Furthermore, inappropriate selection of cultivars in agricultural production of *Zanthoxylum* may cause economic losses to farmers (Feng et al., 2017). Chaotic use of *Zanthoxylum* medicinal materials also directly affects the safety and efficacy of clinical medications. Therefore, reliable and distinguishable genetic markers are urgently needed for the healthy development of the *Zanthoxylum* industry. Although *Zanthoxylum* is widely distributed in China, the systematic breeding of *Zanthoxylum* varieties has not been carried out on a large scale. The classification and naming of the existing *Zanthoxylum* varieties are based on historical, customary names inherited by the local people and lack an authoritative and unified naming.

To solve the problem of *Zanthoxylum* species identification, Li et al., developed simple sequence repeats (SSRs) markers for the chloroplast (cp) genome of *Z. bungeanum*. Li et al. (2019) showed that SRs can be used for genetic diversity analysis among different *Zanthoxylum* species and used cpSSRs to distinguish between *Z. bungeanum*, *Z. armatum*, and *Z. piperitum*. Feng et al. (2017) developed SSR markers to identify *Z. bungeanum* based on transcriptome data. Wang et al. (2016) used the internal transcribed spacer 2 (ITS2) to identify *Zanthoxylum* plants and found that ITS2 could not completely distinguish all of the *Zanthoxylum* species they selected. However, using the combined barcode of ITS2 and *psbA-trnH* derived from the cp genome, five species of *Zanthoxylum* were distinguished (Wang et al., 2016). This result is consistent with the results of Xin-Ye et al. (2014). The research on molecular markers of *Zanthoxylum* is confined to a few species in some regions only. We are committed to annotating further *Zanthoxylum* complete cp genomes to compare variation and discover additional molecular markers that are applicable to a wider range of *Zanthoxylum* for identification and breeding. The cp genome is characterized by a typical quadripartite structure that contains a pair of inverted repeat (IR) regions separated by a large single-copy (LSC) and a small single-copy (SSC) region (Bendich, 2004). A full cp genome is a valuable resource of information for studying plant taxonomy, phylogenetic reconstruction, and historical biogeographic inference (Liu et al., 2018). The plant cp genome, a research hotspot for screening DNA barcoding sequences, can also be used as a super-barcode for phylogenetic and species identification studies (Jansen et al., 2007). The use of cp genomes to solve the problem of classification of related species is of enormous significance for species identification of herbal medicine and the entire plant community.

In this study, we sequenced and assembled six *Zanthoxylum* whole cp genomes and combined this data with eight previously published *Zanthoxylum* cp genomes to perform a comprehensive analysis, including genome features, repeats, selective pressures, divergence hotspots, and phylogenetic relationships. Our goals in this study were to: (1) present the complete cp genome sequence of six new assembled *Zanthoxylum* species and compare the global structures with another eight previously published *Zanthoxylum* species; (2) examine variations of long repeat sequence and SSRs in 14 *Zanthoxylum* cp genomes; (3) identify divergence hotspots as potential genetic markers for DNA barcoding; and (4) reconstruct the phylogeny of *Zanthoxylum* species using protein-coding sequences of cp genomes and infer their phylogenetic location within *Rutaceae*.

## Materials and Methods

### DNA Extraction and Sequencing

The plant materials of *Z. piasezkii*, *Z. armatum*, *Z. motuoense*, and *Z. oxyphyllum were* collected from Nyingchi (Tibet, China); *Z. multijugum* and *Z. calcicola* were obtained from Kunming (Yunnan, China). Fresh, healthy leaves were directly dried with silica gel after collection. Total genomic DNA was isolated using a modified CTAB method (Li et al., 2013). The DNA integrity and concentration were measured using agarose gel electrophoresis and a NanoDrop 2000 Spectrophotometer (Thermo Scientific, Carlsbad, CA, United States). Purified DNA was randomly sheared into fragments using physical methods. Paired-end reads (150 bp) were generated on an Illumina HiSeq X 10 System (San Diego, CA, United States). Total genomic DNAs were also sent to BGI (Shenzhen, China) for library (400 bp) preparation for genome skimming sequencing. Paired-end (150 bp) sequencing was conducted on the Illumina HiSeq X-10 platform, generating ∼2 Gb data per sample. Low-quality sequences were filtered by NGS QC Toolkit v2.3.333 (Patel and Jain, 2012) with Q30 (base Phred quality score of ≥ 30) was used to obtain high-quality reads.

### cp Genome Assembly and Annotation

We assembled the chloroplast genomes with NOVOPlasty (Dierckxsens et al., 2017) using clean data, with parameters of K-mer (33), and annotated them with GeSeq^1^ (Tillich et al., 2017), coupled with manually edited start and stop codons in Geneious 11.1.4 software (Kearse et al., 2012). The *Z. bungeanum* cp genome sequence (NCBI Accession number: NC031386) was used as a reference. The annotation results were checked using DOGMA^2^ (Wyman et al., 2004) and CpGAVAS (Liu et al., 2012). In addition, all tRNA genes were further verified using tRNAscan-SE v1.21 (Brooks and Lowe, 2005). The boundaries of LSC, SSC, IRa, and IRb were determined through local BLAST software. Finally, the Organellar Genome DRAW tool^3^ (Lohse et al., 2013) was used to draw the circular gene maps of the *Zanthoxylum* cp genome.

### Comparative Genomic Analysis and Molecular Marker Identification

IR expansion and contraction in the cp genomes of the 14 *Zanthoxylum* species were detected using IRscope (Amiryousefi et al., 2018). The nucleotide diversity (Pi) of coding and non-coding regions was extracted (Zhang et al., 2020) and then computed with DnaSP (Rozas et al., 2003). The variable, parsimony informative, conserved sites of DNA barcode sequences were identified using DnaSP software (Rozas et al., 2003).

### Repeat Sequencing Analysis

MISA^4^ was used to identify SSRs in each species with the minimum numbers of repeats set to 8, 5, 3, 3, 3, and 3 for mono-, di-, tri-, tetra-, penta-, and hexanucleotides, respectively (Thiel et al., 2003). The long repetitive sequences containing forward, palindromic, reverse, and complementary repeats were analyzed using the software REPuter^5^ with a 30 bp minimum repeat size and a Hamming distance of 3 (Kurtz et al., 2001).

### Evolutionary and Phylogenetic Analysis

We used the KaKs calculator program (Zhang et al., 2006) with the NG model to calculate the rates of non-synonymous substitutions (Ka), synonymous substitutions (Ks), and their ratio (Ka/Ks). When Ks = 0, the value cannot be computed and was represented by ^∗^. When Ka = 0 and Ks = 0, the value was represented by NaN. The *Sapindales* species *Z. bungeanum* (NC031386.1) was used as a reference.

The genome sequences of 20 plastomes of the *Rutaceae* species were downloaded from the National Center for Biotechnology Information Search database (Supplementary Table 5), and six newly assembled *Zanthoxylum* cp genomes were used to reconstruct phylogenetic relationships; *Xylocarpus rumphii* (NC038199.1) was used as the outgroup in the phylogenetic analysis. A total of 76 protein-coding genes (Supplementary Table 6), shared by the cp genomes of 30 *Rutaceae* species, were selected to construct maximum likelihood (ML) and Bayesian trees. A total of 76 gene sequence alignments were deposited into MAFFT 7.0 (Osaka University, Suita, Japan; Katoh and Standley, 2013) and were adjusted manually where necessary. Phylogenetic trees were constructed using IQ-TREE (Nguyen et al., 2015) and MrBayes 3.2.6 software (Ronquist et al., 2012; Zhang et al., 2020). The Bayesian Inference tree was constructed under the GTRGAMMA model (two parallel runs, 2,000,000 generations), with the initial 25% of sampled data discarded as burn-in. We selected the GTRGAMMA model of nucleotide substitution for ML analysis (Nguyen et al., 2015).

## Results

### Characterization of cp Genomes in *Zanthoxylum* Species

**FIGURE 1:**
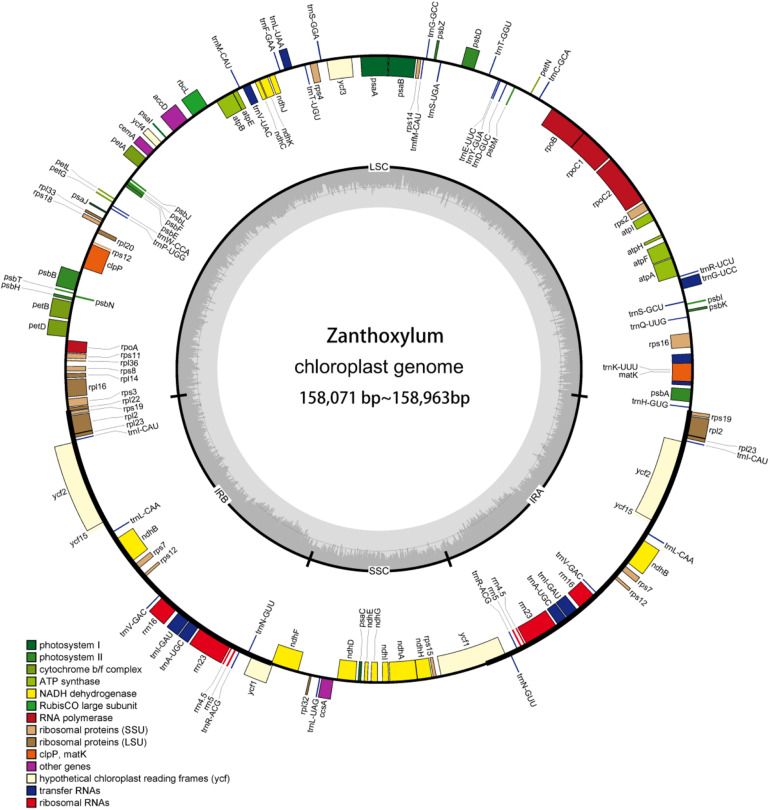
cp genome map of *Zanthoxylum.* Genes in the circle are transcribed clockwise, while the rest are transcribed counterclockwise. Dark gray shading in the inner circle indicates the GC content.

The cp genomes of *Z. piasezkii* (2.1 Gb), *Z. armatum* (2.5 Gb), *Z. motuoense* (1.9 Gb), *Z. oxyphyllum* (2.3 Gb), *Z. multijugum* (2.1 Gb), and *Z. calcicola* (2.2 Gb) were sequenced with approximately 2.0 Gb of paired-end reads, respectively. We obtained the clean reads by removing adaptors and low-quality read pairs. The recovered clean reads for *Z. piasezkii*, *Z. armatum*, *Z. motuoense*, *Z. oxyphyllum*, *Z. multijugum*, and *Z. calcicola* were 1,604,681, 986,037, 804,684, 1,135,946, 1,267,505, and 997,026, respectively (Supplementary Table 1). We obtained complete cp genome maps (Figure 1) of *Z. piasezkii*, *Z. armatum*, *Z. motuoense*, *Z. oxyphyllum*, *Z. multijugum*, and *Z. calcicola* through *de novo* genome sequencing and assembly with the reference *Z. bungeanum* (NC031386) genome. The coverage depth of the final assembled chloroplast genome ranged from 104.2X (*Z. motuoense*) to 322.4X (*Z. piasezkii*) (Supplementary Table 1).

The cp genomes of the 14 *Zanthoxylum* species, their sizes, GC content, number of genes, and other information are shown in Supplementary Table 1. The 14 *Zanthoxylum* cp genomes ranged in size from 158,071 bp for *Z. madagascariense* to 158,963 bp for *Z. schinifolium* (Figure 1 and Supplementary Table 1). The differences between the lengths of the *Zanthoxylum* cp genomes were no greater than 892 bp (Supplementary Table 1). The 14 *Zanthoxylum* cp genomes displayed a typical quadripartite structure, consisting of a pair of IRs (26,955–27,679 bp) separated by one LSC (85,340–86,528 bp) and one SSC (17,526–18,301 bp) region.

The cp genomes of the *Zanthoxylum* species were shown to contain 132 genes, including 87 protein-coding, 37 tRNA, and 8 rRNA genes (Table 1 and Supplementary Table 1). There were 18 duplicated genes, including 4 rRNA genes, and 14 other genes (*ycf2*, *ycf15*, *trnV-GAC*, *trnR-ACG*, *trnN-GUU*, *trnI-CAU*, *trnL-CAA*, *trnA-UGC*, *rps7*, *rps12*, *rps19*, *rpl2*, *rpl23*, and *ndhB*), were repeated once. There were 16 genes with one intron, including 10 coding genes (*ndhA, ndhB, rps12, rps16, rpoC1, atpF, petB, petD, rpl16*, and *rpl2*) and 6 tRNAs (*trnK-UUU, trnL-UAA, trnV-UA, trnI-GAU, trnA-UGC*, and *trnG-UCC*). Two coding genes (*ycf3* and *clpP*) had 2 introns (Table 1). Six newly assembled cp genomes with gene annotations have been submitted to NCBI under GenBank accession numbers MT990979 for *Z. piasezkii*, MT990984 for *Z. armatum*, MT990981 for *Z. motuoense*, MT990980 for *Z. oxyphyllum*, MT990982 for *Z. multijugum*, and MT990983 for *Z. calcicola.*

**TABLE 1:** List of annotated genes in *Zanthoxylum* chloroplast genomes.

Gene group	Gene name
Ribosomal RNAs	*rrn16*(2), *rrn23*(2), *rrn4.5*(2), *rrn5*(2)
Transfer RNAs	trnA-UGC*(2), *trnC-GCA, trnD-GUC, trnE-UUC*, trnF-GAA, trnfM-CAU, trnG-UCC*, trnG-GCC, *trnH-GUG, trnI-CAU*(2), trnI-GAU*, trnK-UUU*, *trnL-CAA*(2), trnL-UAA*, trnL-UAG, trnM-CAU, *trnN-GUU*(2), *trnP-UGG, trnQ-UUG*, *trnR-ACG*(2), *trnR-UCU, trnS-GCU, trnS-GGA*, *trnS-UGA, trnT-GGU, trnT-UGU, trnV-GAC*(2), trnV-UAC*, trnW-CCA, trnY-GUA
Proteins of small ribosomal subunit	rps16*, rps2, rps14, rps15, rps4, rps7(2), rps18, rps12* (2), *rps11, rps8, rps3, rps19*(2)
Proteins of large ribosomal subunit	rpl33, rpl20, rpl36, rpl14, rpl16*, rpl22, rpl2* (2), *rpl23*(2), *rpl32*
Subunits of RNA polymerase	rpoC2, rpoC1*
Photosystem I	*psaB, psaA, psaI, psaJ, psaC*
Photosystem II	*psbA, psbB, psbD, psbE, psbF, psbH, psbI*, *psbJ, psbK, psbL, psbM, psbN, psbT, psbZ*
Cytochrome b/f complex	petA, petB*, petD*, petG, petL, petN
Subunits of ATP synthase	atpA, atpB, atpE, atpF*, atpH, atpI
Protease	clpP**
Large subunit of rubisco	*rbcL*
NADH dehydrogenase	ndhA*, ndhB*(2), *ndhC, ndhD, ndhE, ndhF*, *ndhG, ndhH, ndhI, ndhJ, ndhK*
Maturase	*matK*
Envelope membrane protein	*cemA*
Acetyl-CoA carboxylase	*accD*
Synthesis gene	*ccsA*
Open reading frames (ORF, ycf)	*ycf1, ycf2*(2), ycf3**, ycf4, ycf15(2),

*Indicates gene with one intron and **indicates gene with two introns. (× 2) indicates that the number of the repeat unit is 2. The rps12 gene is a trans-spliced gene.

### Contraction and Expansion of IRs

**FIGURE 2:**
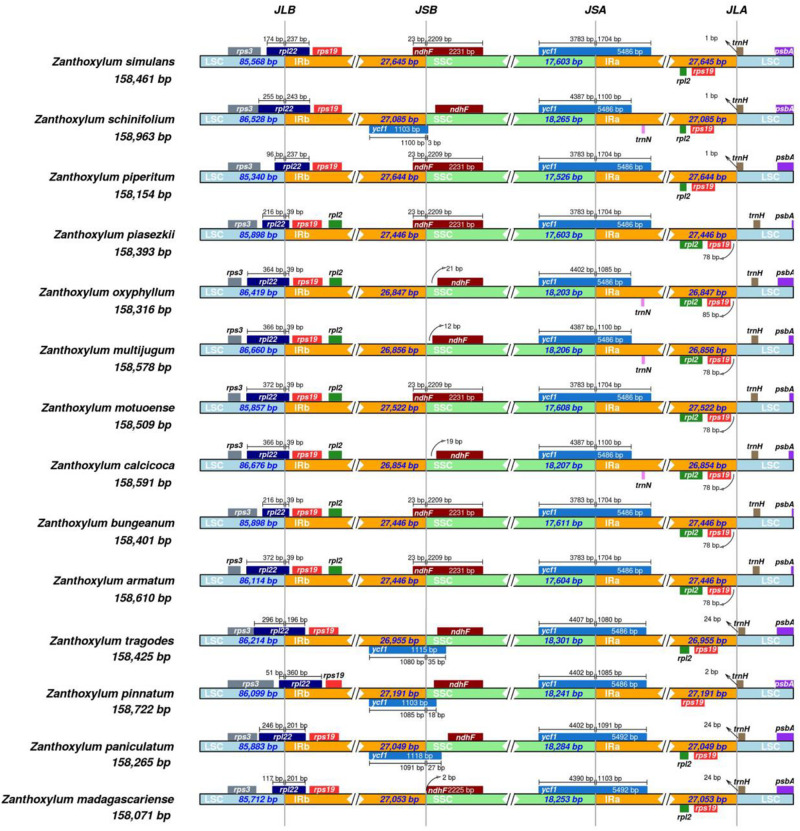
Comparison of the junctions of the LSC, SSC, and IR regions in the cp genomes of 14 *Zanthoxylum* species. JLB denotes the LSC/IRb junction, JSB denotes the SSC/IRb junction, JSA denotes the SSC/IRa junction, and JLA denotes the LSC/IRa junction.

The contraction and expansion of IR regions is the main contributor to the size variation in cp genomes and alters the evolutionary rate of the cp genome (Zhang et al., 2013). We compared the IR boundaries in 14 *Zanthoxylum* species and found that the IR boundary regions varied slightly (Figure 2). The IRa/LSC boundaries were located downstream of the *rps19* gene. The *ycf1* gene crossed over the IRa/SSC border and extended into the IRa region; the length of the ycf1 gene extending into the IRa region ranged from 1,085 to 1,704 bp. At the IRb/SSC border, 23 bp of the *ndhF* gene was located within the IRb while the remainder was situated in the SSC regions, except in *Z. oxyphyllum, Z. multijugum, Z. calcicoca, Z. pinnatum, Z. madagascariense, Z. schinifolium, Z. paniculatum*, and *Z. tragodes*, where the *ndhF* gene was fully present within the SSC region, indicating that these species may have a closer genetic relationship.

### Long Repeat Analysis

**FIGURE 3:**
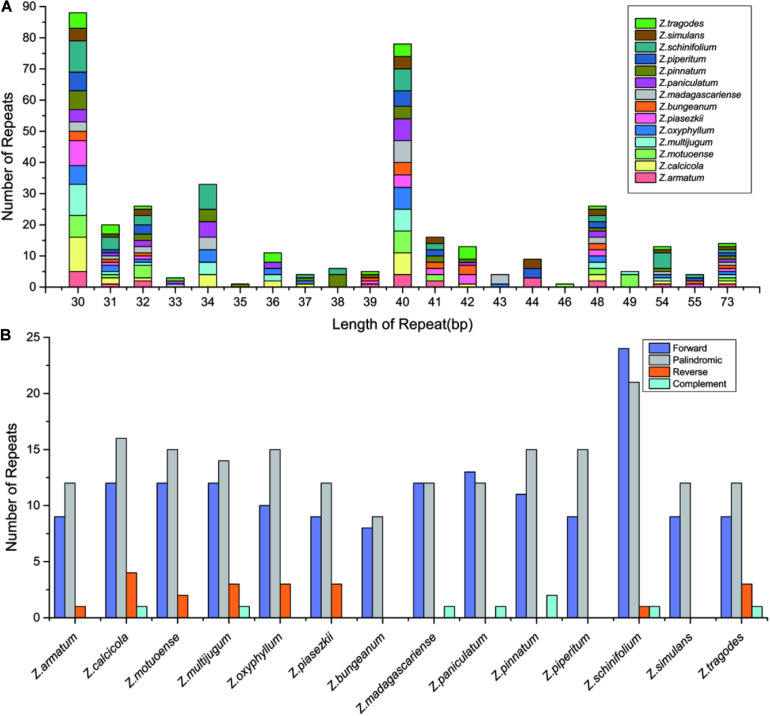
The number of long repeats in the whole cp genome sequence of the 14 *Zanthoxylum* species. **(A)** Frequency of repeats more than 30 bp long. **(B)** Frequency of repeat type.

Repeat sequences in *Zanthoxylum* cp genomes were detected by REPuter, with the criterion of a copy size of 30 bp or longer. A total of 379 long repeats consisting of 159 forward repeats, 192 palindromic repeats, 20 reverse repeats, and 8 complement repeats were detected (Figure 3 and Supplementary Table 2). These long repeats ranged from 30 to 73 bp in length. The long repeat length of 30 bp was found the most frequently and existed in all 14 *Zanthoxylum* cp genomes (Figure 3A and Supplementary Table 2). Long repeat lengths of 35 and 46 bp were found the least often and only existed in *Z. pinnatum* and *Z. motuoense* cp genomes, respectively (Figure 3A and Supplementary Table 2). Among the 14 *Zanthoxylum* species, *Z. schinifolium* had the largest number of long repeats with 47, and *Z. bungeanum* had the smallest number of long repeats with 17 (Figure 3B and Supplementary Table 2). The number of forward repeats varied between 8 (*Z. bungeanum*) and 24 (*Z. schinifolium*), and the number of palindromic repeats varied from 9 (*Z. bungeanum*) to 21 (*Z. schinifolium*) (Figure 3B and Supplementary Table 2). Reverse repeats did not exist in all *Zanthoxylum* species; *Z. calcicola* had the most reverse repeats with 4, and *Z. bungeanum*, *Z. madagascariense*, *Z. paniculatum*, *Z. pinnatum*, *Z. piperitum*, and *Z. simulans* did not have any reverse repeats. There was only one complement repeat in *Z. calcicola*, *Z. multijugum*, *Z. madagascariense*, *Z. paniculatum*, *Z. schinifolium*, and *Z. tragodes* cp genomes, *Z. pinnatum* had two complement repeats, and the remainder of the *Zanthoxylum* cp genomes did not have complement repeats (Figure 3B and Supplementary Table 2).

### SSRs Analysis

**FIGURE 4:**
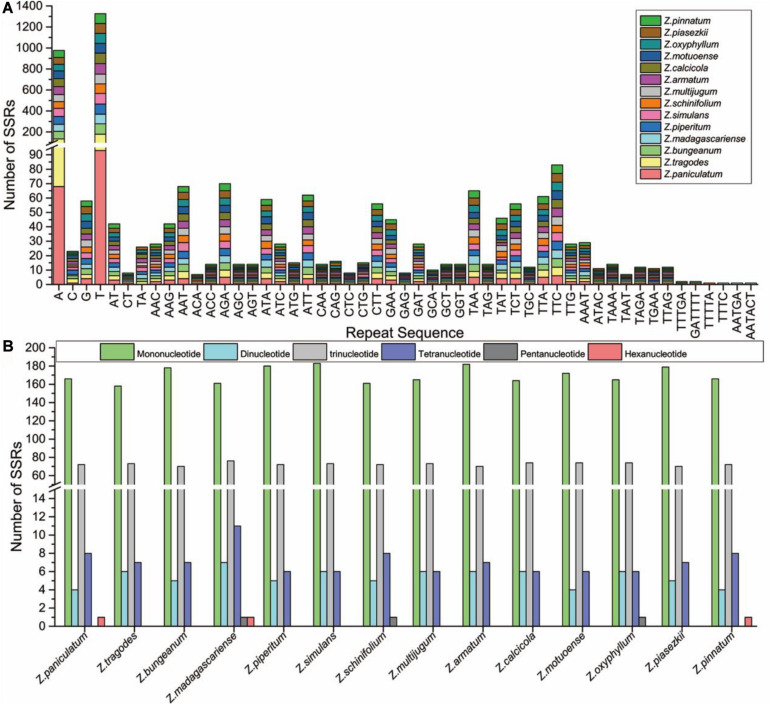
Analysis of SSRs in the 14 *Zanthoxylum* cp genomes. **(A)** Frequency of common motifs in the 14 *Zanthoxylum* cp genomes. **(B)** Number of different SSR types detected in the 14 *Zanthoxylum* cp genomes.

SSRs are important genetic markers to identify closely related species (Zhao et al., 2020; Zheng et al., 2020). Here, SSRs in the cp genomes of 14 *Zanthoxylum* species were detected using MISA software. The number of SSRs in the 14 *Zanthoxylum* species ranged from 244 (*Z. tragodes*) to 268 (*Z. simulans*); no significant differences were found in SSR numbers in the 14 *Zanthoxylum* species (Figure 4A and Supplementary Table 3). In our study, mononucleotide to tetranucleotide SSRs were found in all species. Pentanucleotide repeats were found in *Z. madagascariense, Z. schinifolium*, and *Z. oxyphyllum* cp genomes, and hexanucleotide repeats were also found in *Z. paniculatum* and *Z. madagascariense* (Figure 4B and Supplementary Table 3). Among these SSRs, mononucleotide repeats were the most common (Figures 4A,B). Only a small proportion consisted of dinucleotide, trinucleotide, tetranucleotide, pentanucleotide, and hexanucleotide repeat motifs (Figures 4A,B and Supplementary Table 3). These newly detected SSRs will contribute to the development of genetic markers for the *Zanthoxylum* species in future studies.

### Divergence Hotspots

**FIGURE 5:**
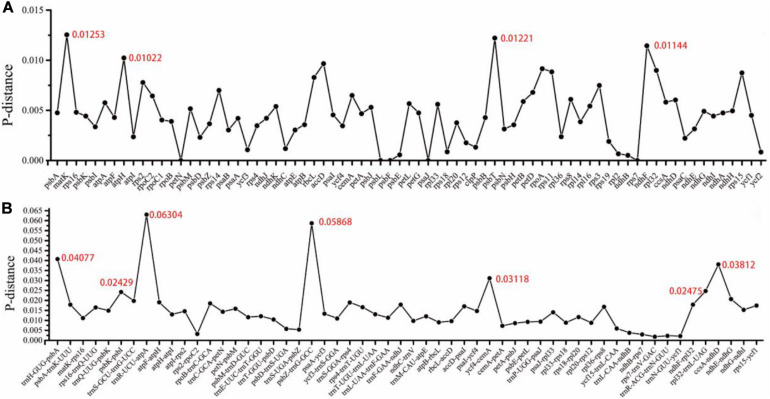
Nucleotide variability (Pi) values were calculated in the 14 *Zanthoxylum* cp genomes. **(A)** Protein-coding genes. **(B)** Intergenic regions. These regions were arranged according to their locations in the cp genome.

We analyzed the nucleotide diversity (Pi) values to measure the divergence level in protein-coding genes and intergenic regions of the 14 *Zanthoxylum* species. The level of sequence divergence among protein-coding genes was more conserved than in intergenic regions. In 76 protein-coding genes, the average Pi value was 0.00456. Based on a considerably higher Pi value of > 0.01, we found four highly variable regions (*psbT*, *ndhF*, *matK*, and *atpH*), and the *matK* gene had the highest divergence value of 0.01253 (Figure 5A). Within the intergenic regions, *trnR-UCU-atpA, psbZ-trnG-GCC, trnH-GUG-psbA, ccsA-ndhD, ycf4-cemA, rpl32-trnL-UAG*, and *psbK-psbI* showed a significantly higher Pi value of > 0.02. The *trnR-UCU-atpA* region had the highest divergence value of 0.06304 (Figure 5B).

We analyzed sequence characteristics such as sequence length range, GC content, and the average number of mutation sites of the seven candidate barcode sequences (Table 2). *ndhF* had the longest sequence length (2,211∼2,229 bp), *ccsA-ndhD* had the shortest sequence length (224∼304 bp). The region with the most average number of mutation sites is *rpl32-trnL-UAG* (13.6%), and the region with the least is *ndhF* (4.4%). From the sequence length and the percentage of parsimony informative sites, *trnH-GUG-psbA, rpl32-trnL-UAG*, and *ccsA-ndhD* contained approximately equal numbers of parsimony informative sites (Table 2).

**TABLE 2:** Basic information of potential DNA barcode sequences.

Sequence name	Length range	GC content	Variable sites	Variable sites	Parsimony informative	Conserved
	(bp)	(%)		(%)	site (%)	sites
*matK*	1,521	36.0∼36.8	71	4.7	2.5	1,450
*ndhF*	2,211∼2,229	34.1∼34.5	98	4.4	2.2	2,131
*ccsA-ndhD*	224∼304	33.8∼38.4	31	10.1	6.5	273
*psbK-psbI*	391∼408	31.3∼33.5	30	7.2	4.1	370
*ycf4-cemA*	635∼654	29.1∼30.2	58	8.5	4.8	604
*rpl32-trnL-UAG*	252∼433	27.0∼31.0	64	13.6	6.6	356
*trnH-GUG-psbA*	307∼492	31.4∼34.5	49	9.6	6.7	429

**FIGURE 6:**
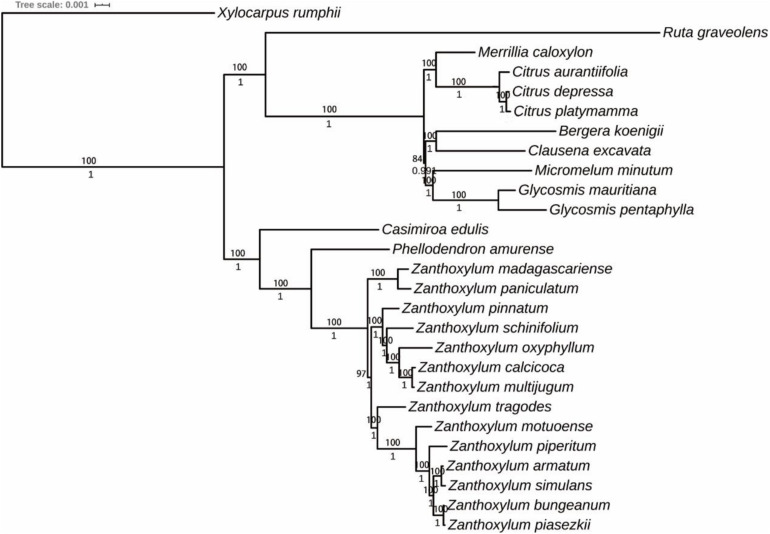
The phylogenetic tree was reconstructed based on 76 protein-coding genes of 27 Rutaceae species cp genomes using IQ-TREE and MrBayes. *Xylocarpus rumphii* was used as the outgroup. Numbers at the nodes denote bootstrap values and posterior probabilities.

Among these high nucleotide diversity regions, we selected seven regions (*matK, ndhF, ccsA-ndhD, psbK-psbI, ycf4-cemA, rpl32-trnL-UAG*, and *trnH-GUG-psbA*) with suitable lengths and low sequence identities as candidate barcode sequences. To evaluate the ability of the seven selected sequences to identify *Zanthoxylum* plants, we constructed the ML tree separately based on each sequence (Supplementary Figure 1). The number of species successfully identified in the ML tree is used to evaluate the resolution power of the sequence (Zheng et al., 2020). When the value of the node is lower than 50, the species on the branch cannot be distinguished (Zheng et al., 2020). *matK* and *rpl32-trnL-UAG* had the same highest resolution power (identification success rate) of 93%, followed by *trnH-GUG-psbA* with 86%, and *ycf4-cemA* with 79%. *CcsA-ndhD* had the lowest resolution power of 57% (Table 3). Additionally, we examined the tree topology of the constructed ML trees based on each region (Figure 6). The tree topology of the constructed ML tree based on the *matK* gene is closest to the evolutionary tree, which was constructed based on the protein-coding genes (Figure 6 and Supplementary Figure 1).

**TABLE 3:** Evaluation of the identification power of seven regions in *Zanthoxylum.*

Species	Bootstrap values of seven regions on ML trees						
	*matK*	*ndhF*	*ccsA-ndhD*	*psbK-psbI*	*ycf4-cemA*	*rpl32-trnL-UAG*	*trnH-GUG-psbA*
*Z. armatum*	99	79	10	88	83	99	71
*Z. simulans*	99	79	27	88	83	95	71
*Z. piperitum*	41	76	27	93	64	68	100
*Z. bungeanum*	99	99	73	90	100	75	96
*Z. piasezkii*	99	99	73	90	100	75	96
*Z. motuoense*	96	100	87	–	64	68	62
*Z. tragodes*	95	43	93	98	66	–	63
*Z. madagascariense*	100	99	43	43	38	98	100
*Z. paniculatum*	100	99	43	70	66	98	100
*Z. pinnatum*	99	19	100	32	49	59	–
*Z. schinifolium*	90	21	–	32	49	59	16
*Z. oxyphyllum*	80	21	100	67	88	93	63
*Z. calcicola*	96	95	92	98	96	100	100
*Z. multijugum*	96	95	92	98	96	100	100
*Identification success rate (%)*	93	71	57	71	79	93	86

**Identification success rate (%) = [(the total number of species - the number of species with bootstrap values below 50%)/the total number of species] × 100% (Zheng et al., 2020).**

### Selective Pressures in the Evolution of Sapindales

We analyzed the synonymous and non-synonymous change rates of 68 protein-coding genes in *Sapindales* (Supplementary Table 4). Eight genes (*ccsA*, *cemA*, *matK*, *psaI*, *psbK*, *psbM*, *rps12*, and *rps16*) were identified under positive selection (Ka/Ks ratio > 1; Supplementary Table 4). This shows that although *Sapindales* face relatively weak selection pressure, some are undergoing essential adaptations to their environment. Among the eight genes, *rps16*, *psbK*, *matK*, *ccsA*, *cemA*, and *rps12* showed high rates for one species. The genes *psbM* and *psaI* presented high rates for two and five species, respectively.

### Phylogenetic Analysis Within Rutaceae

A total of 30 *Rutaceae* cp genomes were selected to perform phylogenetic analysis. *Xylocarpus rumphii* (NC038199.1) was used as the outgroup. The phylogenetic tree was constructed using the ML and Bayesian inference (BI) methods and resulted in similar phylogenetic trees based on 76 protein-coding sequences. Seven *Subgen. Fagara* and five *Subgen. Zanthoxylum* species were clustered together to form a single clade, which is consistent with the record in Flora of China (Huang, 1997). However, it is noteworthy that *Z. madagascariense* and *Z. paniculatum* clustered together to form a single clade and then gather with other *Zanthoxylum* species. The traditional classification system of *Zanthoxylum* mainly relies on the differentiation of calyx and petals. The existing classification of *Zanthoxylum* may be imperfect. Therefore, we speculate that *Z. madagascariense* and *Z. paniculatum* may constitute a new subgenus.

## Discussion

In the present study, we sequenced and annotated the cp genomes of six *Zanthoxylum* species, compared genomic features among the species of *Zanthoxylum*, identified SSR, tandem repeats and suitable polymorphic loci for designing of suitable molecular markers. Our results have laid the foundation for future studies on the molecular identification of *Zanthoxylum* species.

The cp genome of most angiosperm species contains 74 protein-coding genes, while a few species contain another five protein-coding genes (Millen et al., 2001; Bock and Knoop, 2012). In this study, the 14 *Zanthoxylum* cp genomes contain 87 protein-coding genes (79 unigenes were protein-coding), 37 tRNA genes, and 8 rRNA genes, which is similar to *Citrus* (Carbonellcaballero et al., 2015). Although there is one less protein-coding gene in *Z. piperitum* compared with other *Zanthoxylum*, after careful proofreading, we found that the original author missed an annotation for the *rps12* gene (Lee et al., 2015).

Cp genomes are typically 120–160 kb in size since IR regions expand and contract (Wicke et al., 2011). The cp genomes of the 14 *Zanthoxylum* are ∼158 kb, and the length does not change significantly. Although IR boundary regions have no significant changes in *Zanthoxylum*, we found most of the *ndhF* genes of *Subgen. Zanthoxylum* have 23 bp located in the SSC regions, which indicates that the location information of the genes in the IR region can indicate the distance between species to a certain extent.

In addition to identifying closely related species, the variation in SSR copy numbers in the cp genome is an efficient marker for the study of plant population genetics, polymorphism investigations, and evolutionary history (Xue et al., 2012, 2019; He et al., 2012; Wheeler et al., 2014). Li et al. (2019) developed SSR markers derived from cp genomes that can effectively distinguish *Z. bungeanum*, *Z. armatum*, and *Z. piperitum* and used the SSR markers to analyze the genetic diversity among different species of *Zanthoxylum*. The number of Poly (A)/(T) SSRs in the *Zanthoxylum* cp genome is much greater than that of poly(G)/(C), which is consistent with the results of previous studies (Xue et al., 2019; Zhao et al., 2020). The abundant SSRs we found in the cp genomes of the 14 *Zanthoxylum* species laid the foundation for the identification of assays detecting polymorphisms at the population level of *Zanthoxylum*.

Since the whole cp genome contains abundant mutation sites, it can be used directly as a super barcode for species identification., on the other hand, hypervariable regions can be screened out as potential molecular markers for species identification (Nock et al., 2011; Li et al., 2015). Recently, researchers have successfully identified plants such as Amomum (Cui et al., 2019) and Ligularia (Chen et al., 2018) based on the whole cp genome. Hollingsworth et al., proposed using the *rbcL* + *matK* gene combination derived from the cp genome as the core barcode for land plant identification (Hollingsworth et al., 2009). In this research, we selected seven regions (*matK, ndhF, ccsA-ndhD, psbK-psbI, ycf4-cemA, rpl32-trnL-UAG*, and *trnH-GUG-psbA*) as candidate barcode sequences to identify *Zanthoxylum*. Although the protein-coding genes of cp genomes are relatively conservative and are mainly used for the study of higher classification levels, they also have applications in lower classification levels. The *matK* gene is a good identifier for plants of *Apocynaceae* (Cabelin and Alejandro, 2016), *Dipterocarpaceae* (Hu et al., 2019), and *Juniperus* (Hong et al., 2014). Given the excellent performance of the *matK* gene in the construction of the evolutionary tree of *Zanthoxylum*, we recommend that the *matK* gene be used to reconstruct phylogenetic relationships of *Zanthoxylum* where there is a lack of genomic information. Although the *psbT* gene in *Zanthoxylum* cp genomes has a high Pi value, the length of the *psbT* gene is too short (102 bp) to provide sufficient mutation sites, so we believe that *psbT* is not suitable as a barcode gene. Similarly, the *rpl22* gene has a high Pi value in *Zanthoxylum* cp genomes mainly due to the variation in the *rpl22* gene length, making it difficult to design universal primers and so *rpl22* is not suitable as a barcode gene. Due to less selective pressure, non-coding sequences have higher evolutionary rates than coding regions and so provide more systematically significant information sites (Borsch and Quandt, 2009). *trnH-psbA* and *rpl32-trnL* are often used in genus level and subgenus level relationships, phylogenetic location, and species identification research. In our study, *trnH-psbA* and *rpl32-trnL* had excellent species identification success rates in *Zanthoxylum*. We recommend *matK*, *trnH-psbA*, and *rpl32-trnL* sequences as potential molecular markers for the identification and marker-assisted breeding of *Zanthoxylum*. In previous studies, the construction of *Zanthoxylum* phylogeny was mostly based on SSR markers (Feng et al., 2016), random amplified polymorphic DNA (Hong et al., 2008), sequence-related amplified polymorphism markers (Feng et al., 2015), and single-copy nuclear genes (Feng et al., 2017). The lack of genomic information hinders the accurate evolutionary analysis of *Zanthoxylum* and its related species (Feng et al., 2017). Since the cp genome sequence is relatively conservative and is less affected by paralogous genes such as nuclear genes when constructing phylogenetic trees, it has often been used in angiosperm phylogeny construction and speculation of species evolution history in recent years (Dong et al., 2017; Zhang et al., 2017; Zhao et al., 2020).

In our study, 14 *Zanthoxylum* species were represented with strongly supported phylogenetic trees using ML and BI analysis. The results of our phylogenetic analysis strongly support the genus *Fagara* as a subgenus of *Zanthoxylum*, and proposes the possibility of a new subgenus in *Zanthoxylum. Z. bungeanum* and *Z. schinifolium* recorded in the Chinese Pharmacopeia belong to a different subgenus and are relatively distantly related, consistent with the research of Wang et al. (2016). Overall, the phylogenetic position of *Zanthoxylum* revealed by our phylogenetic tree is more credible than in previous studies given the higher number of cp genomes analyzed in our research.

## Data Availability Statement

The cp genome sequences of Zanthoxylum species were submitted on the National Center for Biotechnology Information (NCBI) and the accession numbers were: MT990979, MT990984, MT990981, MT990980, MT990982, and MT990983.

## Author Contributions

KZ, LL, and XL conceived and designed the work. HQ and JY collected the samples. KZ, LL, and ZZ performed the experiments and analyzed the data. KZ and LL wrote the manuscript. XL and ZL revised the manuscript. All authors have read and agreed to the published version of the manuscript.

## Conflict of Interest

The authors declare that the research was conducted in the absence of any commercial or financial relationships that could be construed as a potential conflict of interest.

## Footnotes

1 https://chlorobox.mpimp-golm.mpg.de/geseq.html

2 http://dogma.ccbb.utexas.edu/

3 https://chlorobox.mpimp-golm.mpg.de/OGDraw.html

4 https://webblast.ipk-gatersleben.de/misa/

5 https://bibiserv.cebitec.uni-bielefeld.de/reputer

## Supplementary Material

Supplementary Figure 1 ML trees constructed based on seven regions.

Supplementary Table 1 Summary of the characteristics of the 14 *Zanthoxylum* cp genomes.

Supplementary Table 2 The number of long repeats in the whole cp genome sequence of 14 Zanthoxylum species.

Supplementary Table 3 SSRs comparison of 14 *Zanthoxylum* species.

Supplementary Table 4 The rates of Ka, Ks, and KaKs of 68 genes among 13 Sapindales species.

Supplementary Table 5 List of species used for phylogenetic tree construction.

Supplementary Table 6 List of 76 protein-coding genes used for phylogenetic tree construction.
