# Supplementary figures and images for: Comparative Analyses of Chloroplast Genomes From 14 Zanthoxylum Species: Identification of Variable DNA Markers and Phylogenetic Relationships Within the Genus
Source: Front Plant Sci. 2021 Jan 13;11:605793. doi: 10.3389/fpls.2020.605793 (PMC7838127; doi:10.3389/fpls.2020.605793)

**Supplementary Figure S1.** ML trees based on seven regions.

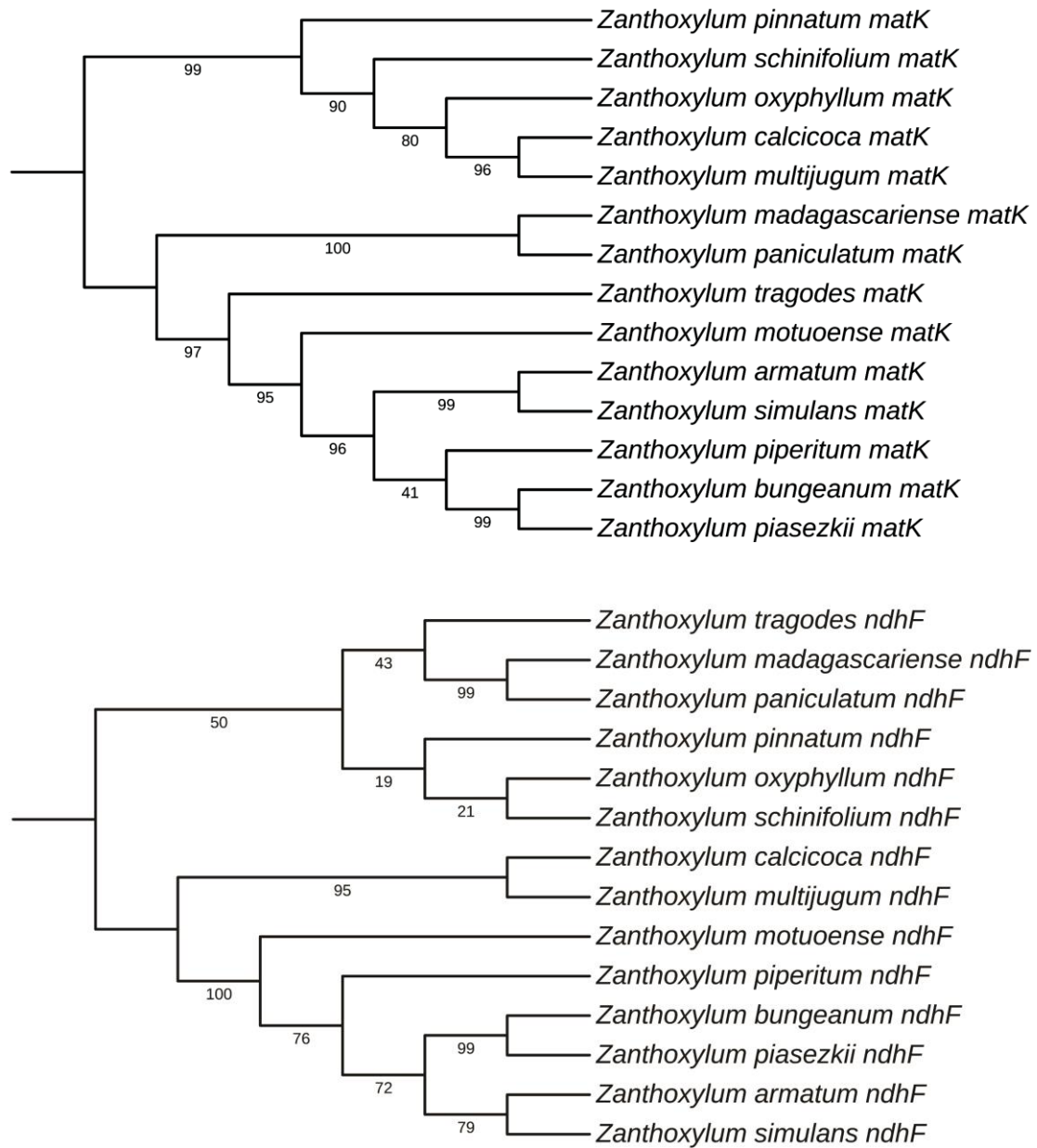

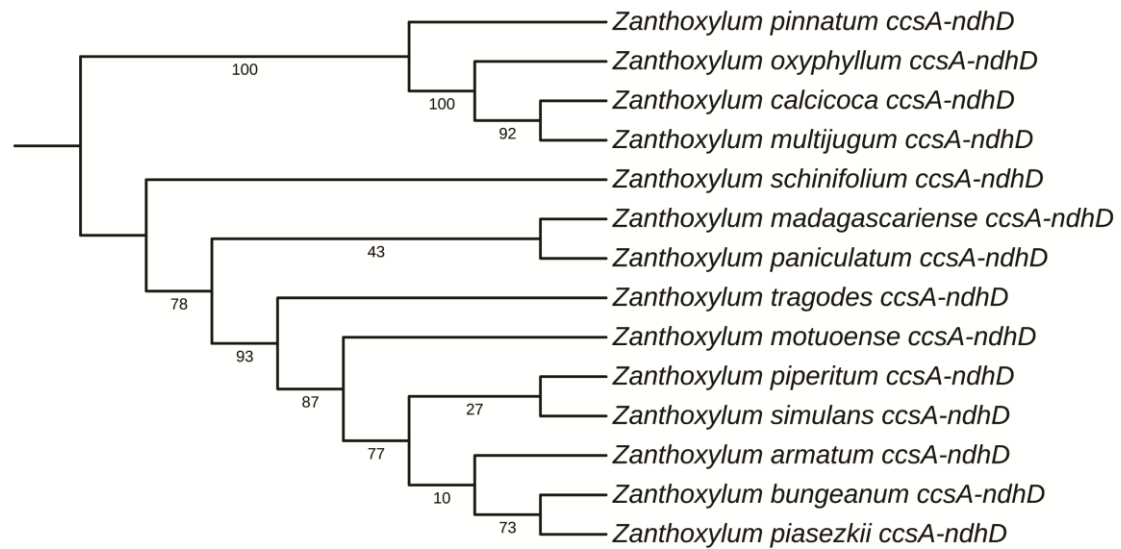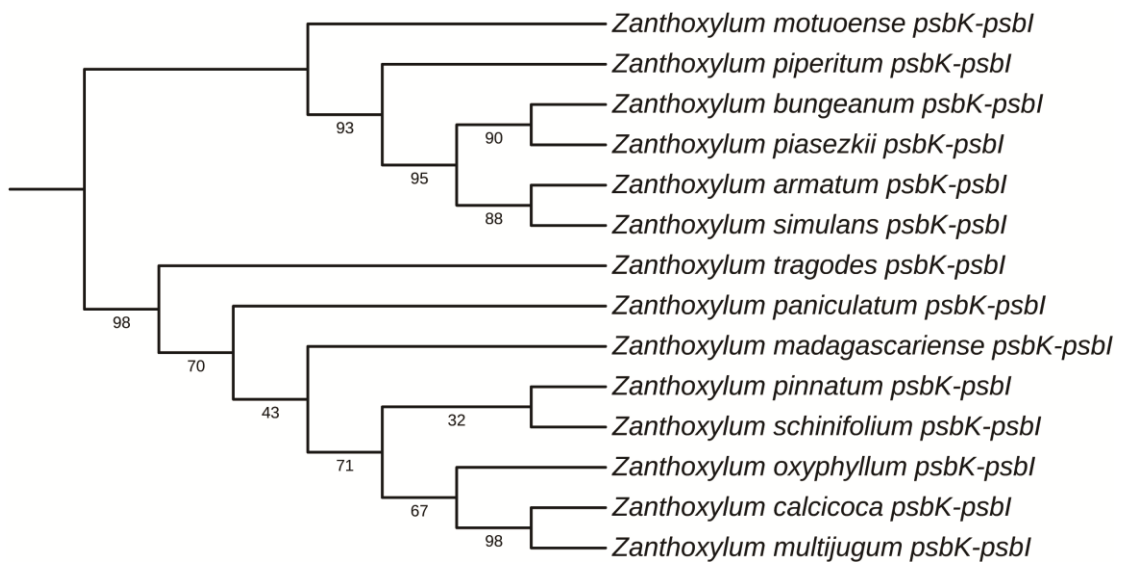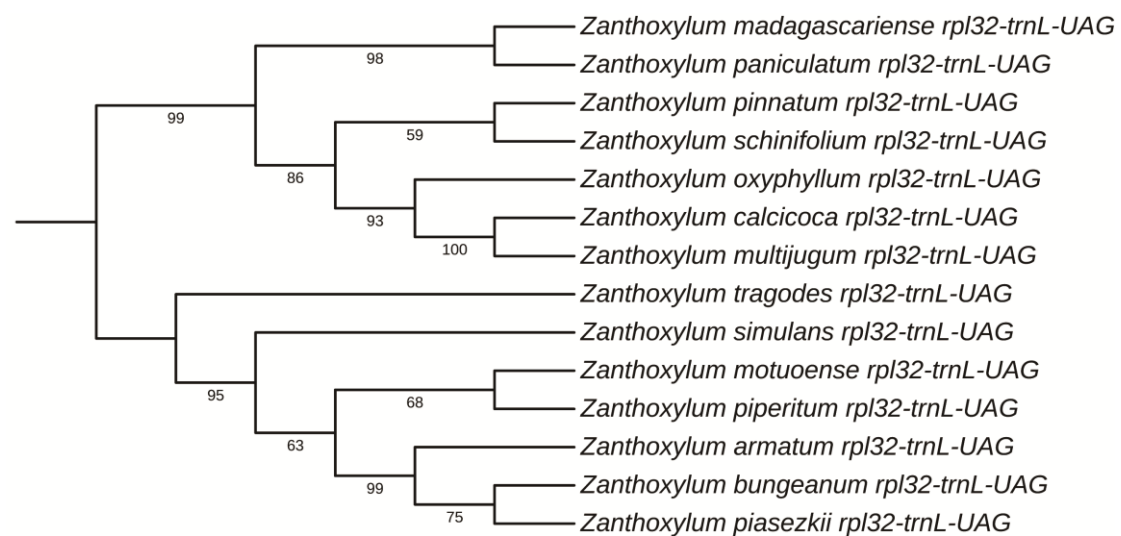

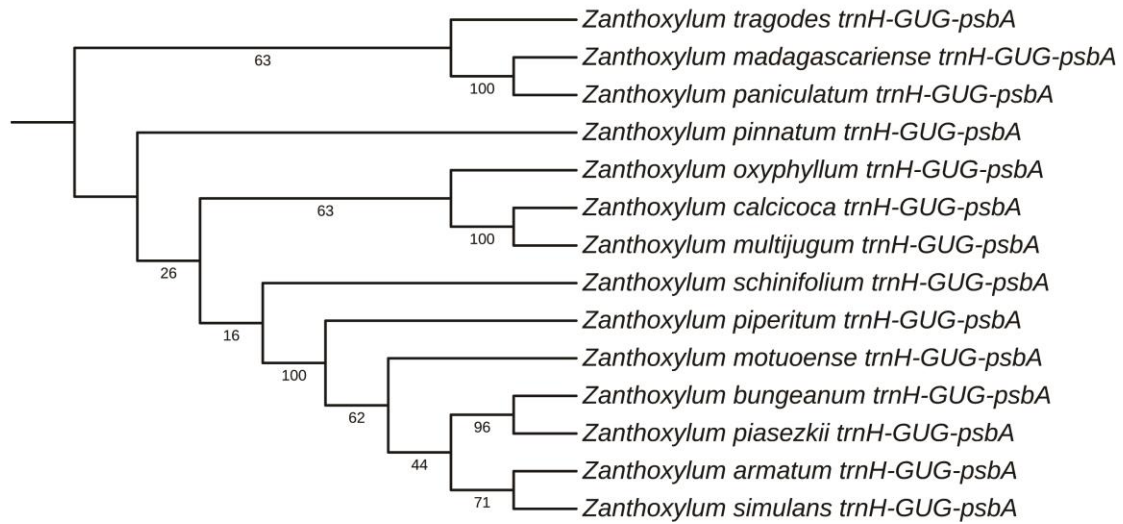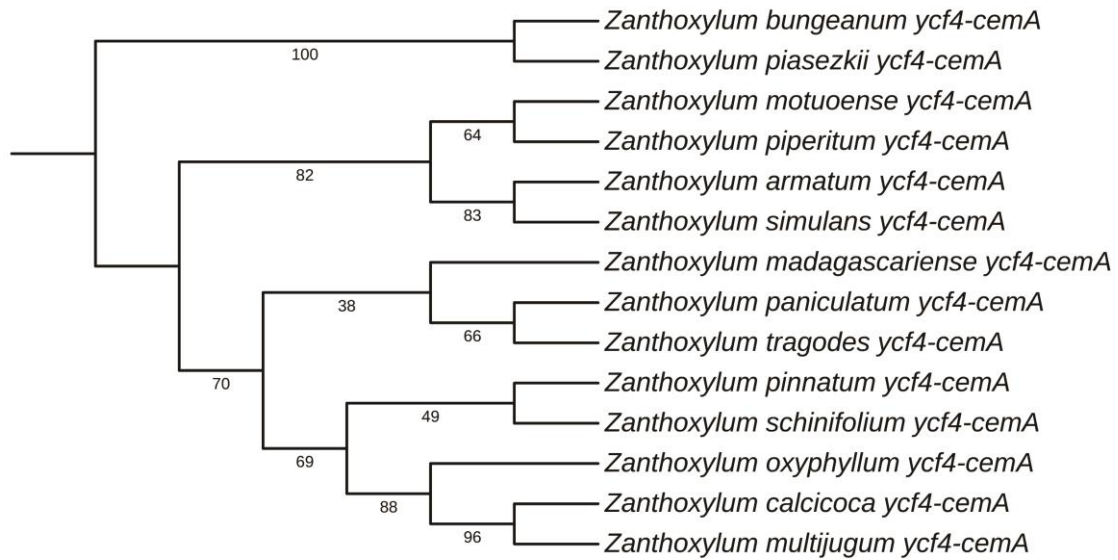

Supplement: Supplementary Figure 1 — ML trees constructed based on seven regions. [file Data_Sheet_1.PDF]
