# Supplementary material for: Comparative Analyses of Chloroplast Genomes From 14 Zanthoxylum Species: Identification of Variable DNA Markers and Phylogenetic Relationships Within the Genus
Source: Front Plant Sci. 2021 Jan 13;11:605793. doi: 10.3389/fpls.2020.605793 (PMC7838127; doi:10.3389/fpls.2020.605793)
Supplement: Supplementary Table 5 — List of species used for phylogenetic tree construction. [file Table_5.DOCX]

**Table S5.** List of species used for phylogenetic tree construction.

|  | Species | GenBank Number |
| --- | --- | --- |
| 1 | *Bergera koenigii* | JF715054 |
| 2 | *Casimiroa edulis* | JF715057 |
| 3 | *Citrus aurantiifolia* | MH373593 |
| 4 | *Citrus depressa* | MH879018 |
| 5 | *Citrus platymamma* | MH286318 |
| 6 | *Clausena excavata* | MK397875 |
| 7 | *Glycosmis mauritiana* | KX279888 |
| 8 | *Glycosmis pentaphylla* | MG836508 |
| 9 | *Merrillia caloxylon* | MK397861 |
| 10 | *Micromelum minutum* | MK795965 |
| 11 | *Phellodendron amurense* | KY679199 |
| 12 | *Ruta graveolens* | MK397854 |
| 13 | *Zanthoxylum bungeanum* | MK397856 |
| 14 | *Zanthoxylum madagascariense* | EU254477 |
| 15 | *Zanthoxylum paniculatum* | KY275182 |
| 16 | *Zanthoxylum pinnatum* | KM201427 |
| 17 | *Zanthoxylum piperitum* | MK330002 |
| 18 | *Zanthoxylum schinifolium* | KY985269 |
| 19 | *Zanthoxylum simulans* | NC027728 |
| 20 | *Zanthoxylum tragodes* | KX774248 |
| 21 | *Zanthoxylum piasezkii* | MT990979 |
| 22 | *Zanthoxylum armatum* | MT990984 |
| 23 | *Zanthoxylum motuoense* | MT990981 |
| 24 | *Zanthoxylum oxyphyllum* | MT990980 |
| 25 | *Zanthoxylum multijugum* | MT990982 |
| 26 | *Zanthoxylum calcicola* | MT990983 |
| 27 | *Xylocarpus rumphii* | MN514858 |
