# Supplementary material for: Comparative Analyses of Chloroplast Genomes From 14 Zanthoxylum Species: Identification of Variable DNA Markers and Phylogenetic Relationships Within the Genus
Source: Front Plant Sci. 2021 Jan 13;11:605793. doi: 10.3389/fpls.2020.605793 (PMC7838127; doi:10.3389/fpls.2020.605793)
Supplement: Supplementary Table 6 — List of 76 protein-coding genes used for phylogenetic tree construction. [file Table_6.DOCX]

**Table S6.** List of 76 protein-coding genes used for phylogenetic tree construction.

|  | Genes |  | Genes |  | Genes |  | Genes |
| --- | --- | --- | --- | --- | --- | --- | --- |
| 1 | *accD* | 20 | *ndhI* | 39 | *psbH* | 58 | *rpoA* |
| 2 | *atpA* | 21 | *ndhJ* | 40 | *psbI* | 59 | *rpoB* |
| 3 | *atpB* | 22 | *ndhK* | 41 | *psbJ* | 60 | *rpoC1* |
| 4 | *atpE* | 23 | *petA* | 42 | *psbK* | 61 | *rpoC2* |
| 5 | *atpF* | 24 | *petB* | 43 | *psbL* | 62 | *rps11* |
| 6 | *atpH* | 25 | *petD* | 44 | *psbM* | 63 | *rps12* |
| 7 | *atpI* | 26 | *petG* | 45 | *psbN* | 64 | *rps14* |
| 8 | *ccsA* | 27 | *petL* | 46 | *psbT* | 65 | *rps16* |
| 9 | *cemA* | 28 | *petN* | 47 | *psbZ* | 66 | *rps18* |
| 10 | *clpP* | 29 | *psaA* | 48 | *rbcL* | 67 | *rps19* |
| 11 | *matK* | 30 | *psaB* | 49 | *rpl14* | 68 | *rps2* |
| 12 | *ndhA* | 31 | *psaC* | 50 | *rpl16* | 69 | *rps3* |
| 13 | *ndhB* | 32 | *psaI* | 51 | *rpl20* | 70 | *rps4* |
| 14 | *ndhC* | 33 | *psaJ* | 52 | *rpl22* | 71 | *rps7* |
| 15 | *ndhD* | 34 | *psbA* | 53 | *rpl23* | 72 | *rps8* |
| 16 | *ndhE* | 35 | *psbB* | 54 | *rpl2* | 73 | *ycf1* |
| 17 | *ndhF* | 36 | *psbD* | 55 | *rpl32* | 74 | *ycf2* |
| 18 | *ndhG* | 37 | *psbE* | 56 | *rpl33* | 75 | *ycf3* |
| 19 | *ndhH* | 38 | *psbF* | 57 | *rpl36* | 76 | *ycf4* |
